# Passive Transfer of Blood Sera from ALS Patients with Identified Mutations Results in Elevated Motoneuronal Calcium Level and Loss of Motor Neurons in the Spinal Cord of Mice

**DOI:** 10.3390/ijms22189994

**Published:** 2021-09-16

**Authors:** Tamás F. Polgár, Valéria Meszlényi, Bernát Nógrádi, Laura Körmöczy, Krisztina Spisák, Kornélia Tripolszki, Márta Széll, Izabella Obál, József I. Engelhardt, László Siklós, Roland Patai

**Affiliations:** 1 Biological Research Centre, Institute of Biophysics, 62 Temesvári krt., 6726 Szeged, Hungary; polgar.tamas@brc.hu (T.F.P.); mesval13@gmail.com (V.M.); bernatnogradi@gmail.com (B.N.); laura.kormoci96@gmail.com (L.K.); spisakkrisztina96@gmail.com (K.S.); 2 Theoretical Medicine Doctoral School, University of Szeged, 97 Tisza Lajos krt., 6722 Szeged, Hungary; 3 Albert Szent-Györgyi Health Centre, Department of Neurology, University of Szeged, 6 Semmelweis u., 6725 Szeged, Hungary; obalizabella@yahoo.com (I.O.); eji48dec9@yahoo.com (J.I.E.); 4 Department of Medical Genetics, University of Szeged, 4/B Szőkefalvi-Nagy Béla u., 6720 Szeged, Hungary; tripolszki.kornelia@med.u-szeged.hu (K.T.); szell.marta@med.u-szeged.hu (M.S.); 5 Dermatological Research Group, Hungarian Academy of Sciences, University of Szeged, 4/B Szőkefalvi-Nagy Béla u., 6720 Szeged, Hungary; 6 Department of Neurology, Aalborg University Hospital, 15 Skovvej Sdr., 9000 Aalborg, Denmark

**Keywords:** amyotrophic lateral sclerosis, passive transfer, blood serum, motoneuronal calcium increase, motoneuronal loss, *C9ORF72*

## Abstract

**Introduction:** Previously, we demonstrated the degeneration of axon terminals in mice after repeated injections of blood sera from amyotrophic lateral sclerosis (ALS) patients with identified mutations. However, whether a similar treatment affects the cell body of motor neurons (MNs) remained unresolved. **Methods:** Sera from healthy individuals or ALS patients with a mutation in different ALS-related genes were intraperitoneally injected into ten-week-old male Balb/c mice (*n* = 3/serum) for two days. Afterward, the perikaryal calcium level was measured using electron microscopy. Furthermore, the optical disector method was used to evaluate the number of lumbar MNs. **Results:** The cytoplasmic calcium level of the lumbar MNs of the ALS-serum-treated mice, compared to untreated and healthy-serum-treated controls, was significantly elevated. While injections of the healthy serum did not reduce the number of MNs compared to the untreated control group, ALS sera induced a remarkable loss of MNs. **Discussion:** Similarly to the distant motor axon terminals, the injection of blood sera of ALS patients has a rapid degenerative effect on MNs. Analogously, the magnitude of the evoked changes was specific to the type of mutation; furthermore, the degeneration was most pronounced in the group treated with sera from ALS patients with a mutation in the *chromosome 9 open reading frame 72* gene.

## 1. Introduction

Amyotrophic lateral sclerosis (ALS) is one of the most common yet still incurable disorders affecting, primarily, the motor nervous system [1]. Historically, ALS patients are sorted into two main categories based on previous involvement in the family. Underlying both familiar (5–10% of all ALS patients) and sporadic cases (90–95% of all ALS patients), not a single pathological process could be identified. Still, several degenerative pathways have been recognized, which, through their complex interactions, result in similar clinical phenotypes [2,3]. The most well-characterized mechanisms include oxidative stress [4], glutamate excitotoxicity [5], proteinopathies [6], mitochondrial dysfunctions [7], neuroinflammation [8,9], disturbance in the calcium homeostasis [10], failure in RNA processing [11] and the axonal transport [12]. Another interesting phenomenon is that these pathological processes are not limited to ALS, but they can contribute to numerous neurological disorders such as Alzheimer’s disease, Parkinson’s disease, Huntington’s disease, and some other neurological diseases as well [13].

The clinical manifestation of ALS starts with fatigue—and as the disease progresses—, muscle weakness, paralysis, and, eventually, death occurs primarily due to respiratory failure [14]. One of the most well-known cellular manifestations of the disease besides the glial scar formation in the lateral tract of the spinal cord is the loss of lower motor neurons (MNs) in the spinal cord and upper MNs in the brainstem and motor cortex [15,16]. Since degeneration and the increasing loss of MNs underlies the progressively developing functional deficit of muscular performance characteristic to ALS, animal models are expected to replicate this feature. Thus, while similarities of the degenerative changes observed in the motor axon terminals isolated either from the patients’ muscle biopsy samples or from different animal models can be used as a measure of the appropriateness of the actual model, based on the pathological findings in the animals’ spinal cord, assumptions can be set up about the ongoing alterations in the living human spinal cord, which, for ethical reasons, is unavailable for sampling. Indeed, increased synaptic vesicle density and increased calcium, which were demonstrated in the motor axon terminals in biopsy samples of ALS patients [17], could also be documented in the neuromuscular synapses of the interosseus muscle of the superoxide dismutase 1 (SOD1) G93A transgenic mouse, modeling ALS [18]. Similar changes of the motor axon terminals could be observed in a passive transfer model of the disease, i.e., after injecting the animals with purified immunoglobulin G (IgG) [19] or whole sera obtained from sporadic ALS patients [20]. In both models, the demonstrated calcium increase in the spinal MNs implied the importance of the elevated calcium in the pathomechanism of the disease. Recently, after injecting the whole sera of patients with identified mutations, similar alteration could be observed in the motor axon terminals of the interosseus muscle of the injected animals [21], suggesting that calcium-mediated processes might be the common denominators of the disease, regardless of the presence of mutations identified in the patients. However, the deleterious effect of the sera from these patients on the perikarya of the MNs has not been documented yet.

Thus, in the present experiment, the same experimental paradigm was applied as in our earlier experiments, i.e., sera from the same patients with identified mutations in the *SOD1*, *C9ORF72*, *sequestosome 1* (*SQSMT1), G2/mitotic-specific cyclin F* (*CCNF*), *never in a mitosis A-related kinase 1* (*NEK1*), *TANK-binding kinase 1* (*TBK1*) and *ubiquilin 2* (*UBQLN2*) genes were injected into mice. Afterward, the number of MNs in the ventrolateral motor pool in the lumbar spinal cord was determined using the optical disector method, and the calcium content of these cells was analyzed electron microscopically.

## 2. Results

### 2.1. Presence of the Human Immunoglobulins in the Spinal Motor Neurons after Passive Transfer of the Amyotrophic Lateral Sclerosis Sera

**Figure 1:**
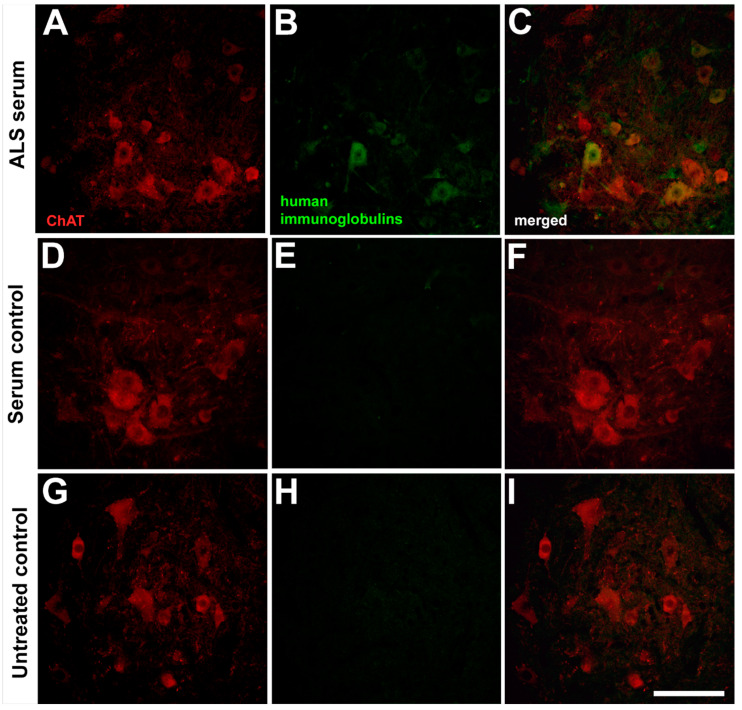
The presence of human immunoglobulins in spinal motor neurons (MNs) can be detected after intraperitoneal injection of sera from amyotrophic lateral sclerosis patients (**C**). The ventrolateral pool of MNs was demonstrated with choline acetyltransferase (ChAT) staining (**A**,**D**,**G**). Presence of immunoglobulins can be detected in sections cut from ALS-treated mice (**B**), and were not present in sections obtained from healthy-serum-treated (**E**) or untreated control animals (**H**). Merged images (**C**,**F**,**I**) show the colocalization of human immunoglobulins with MNs of ALS serum-treated mice only. Scale bar: 200 µm.

Since the whole serum was used for inoculation in this study, the presence of immunoglobulins which are partially responsible for the neuronal degeneration was also visualized using immunohistochemistry [22]. The passive transfer of the ALS sera resulted in the presence of immunoglobulins in MNs after two consecutive days of treatment; however, a similar infiltration of immunoglobulins into the MNs was not visible after treatment with sera from healthy individuals or without any treatment (Figure 1).

### 2.2. Qualitative Morphological Changes of the Lumbar Motor Neurons and Their Calcium Content after Injection of Amyotrophic Lateral Sclerosis Sera

**Figure 2:**
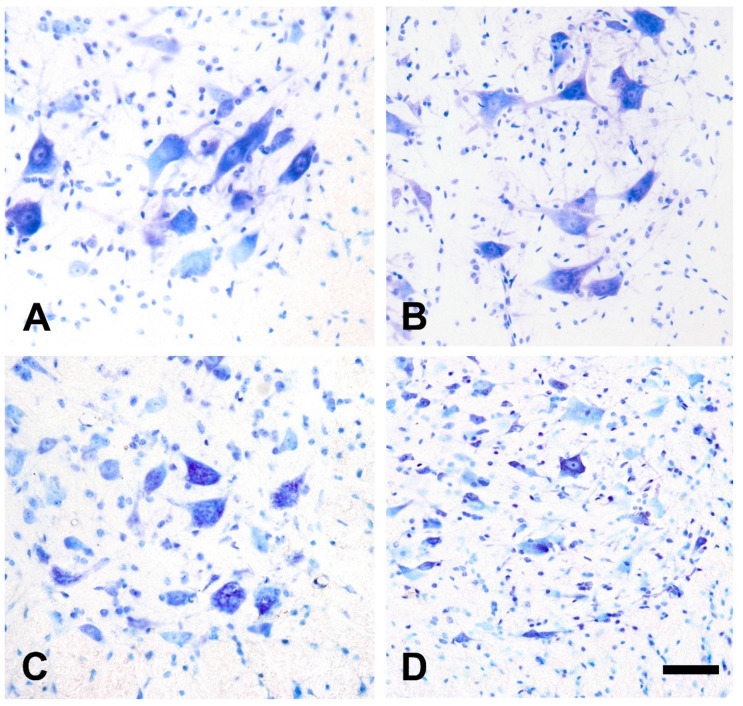
Large cells (>30 µm of characteristic profile size) identified as motor neurons (MNs) are detectable in high number in untreated mice (**A**) or after injection with sera from healthy individuals (**B**). A slight reduction in the number of MNs could be observed after treatment with serum from a patient with *superoxide dismutase 1* pLeu144Phe mutation (**C**). The most prominent neuronal loss was noted in the group treated with sera from patients with *chromosome 9 open reading frame 72* hexanucleotide repeat expansions, (**D**) where almost all MNs diminished. For representative images from other groups, see Figure S1. Scale bar: 100 µm.

The qualitative examination of the ventrolateral MN pool after cresyl violet staining did not show any MN loss in the lumbar spinal cord after a passive transfer from healthy volunteers compared to the untreated animals (Figure 2). However, the passive transfer of ALS sera resulted in a prominent loss of MNs in all groups, but the most notable degeneration could be observed after inoculation with sera from ALS patients with a mutation in the *C9ORF72* gene (Figure 2).

**Figure 3:**
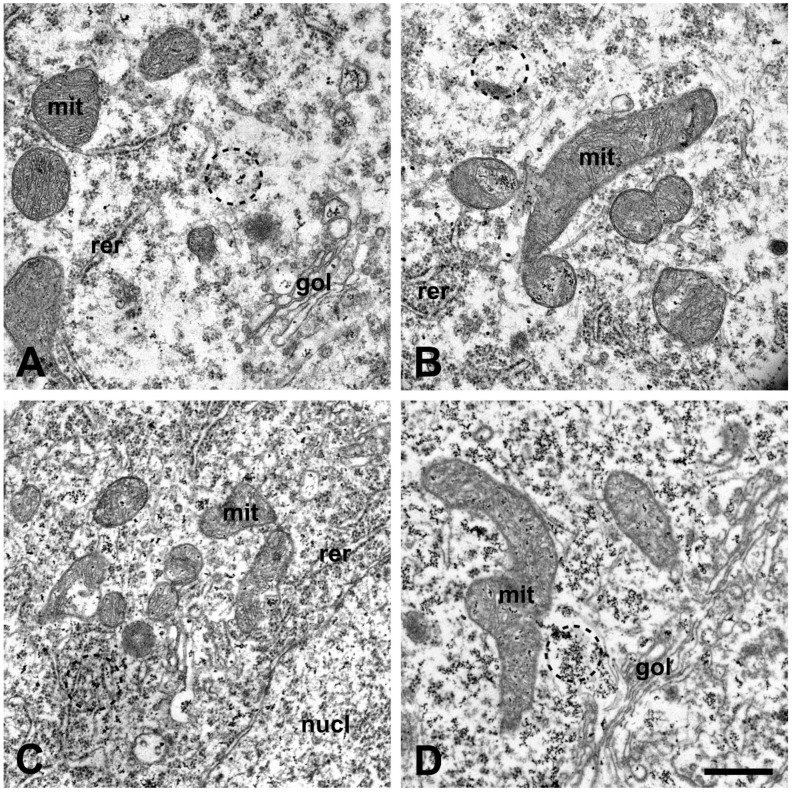
No ultrastructural alterations can be seen in untreated (**A**) and healthy-serum-treated motor neurons (MNs) (**B**). The dilated lumen of the endoplasmic reticulum and mitochondrial cristae structure characterize the micrographs from mice treated with sera from amyotrophic lateral sclerosis (ALS) patients with *superoxide dismutase 1* pLeu144Phe mutation (**C**) and *chromosome 9 open reading frame 72* hexanucleotide repeat expansions (*C9ORF72* HNRs) (**D**). In the motor neurons of ALS-sera-treated mice (**C**,**D**), an elevated number of electron-dense deposits (EDDs), representing the calcium content, can be seen compared to the MNs of untreated (**A**) and healthy-sera-treated animals (**B**). The elevation in the numbers of EDDs was most prominent in the group treated with sera from ALS patients with *C9ORF72* HNRs (**D**). For representative images from the other groups, see Figure S2. Mit: mitochondrion; rer: rough endoplasmic reticulum; gol: Golgi complex; nucl: nucleus. Scale bar: 500 nm.

Electron microscopically, no ultrastructural alteration could be seen in MNs of untreated mice or after treatment with serum from healthy controls. After inoculation with ALS sera, disorganized mitochondrial cristae, furthermore, dilated endoplasmic reticulum and a Golgi complex could be observed in the perikarya of MNs compared to controls (Figure 3A,B). Increased cytoplasmic and mitochondrial calcium, visualized as a larger number of electron-dense deposits (EDDs), could be observed in MNs after treatment with sera from ALS patients with identified mutations (Figure 3C,D). Qualitatively, the elevation in the number of calcium precipitates was most prominent in mice treated with ALS sera from patients with the *C9ORF72* mutation (Figure 3D).

### 2.3. Calcium Level Is Elevated in the Perikarya of Lumbar Motor Neurons after Injection of Amyotrophic Lateral Sclerosis Sera into Mice

**Figure 4:**
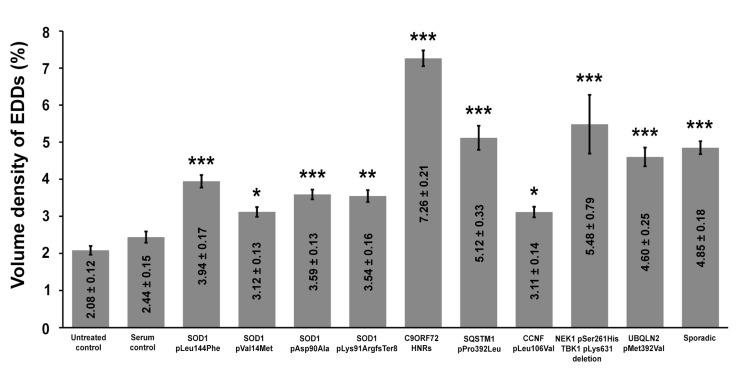
A significant elevation in electron-dense deposits can be observed in motor neurons (MNs) of all amyotrophic lateral sclerosis sera-treated mice. Animals treated with sera from patients with hexanucleotide repeat expansions in the *chromosome 9 open reading frame 72* gene showed the highest increase in intracellular calcium levels. Data values are displayed within the bars, and represented by mean value ± standard error of the mean. Statistical evaluation was determined using one-way analysis of the variance with the Bonferroni post hoc pairwise comparison. *: *p* < 0.05; **: *p* < 0.01; ***: *p* < 0.001.

The intracellular calcium content was expressed as the volume density of the calcium precipitates to analyze changes in the calcium levels. For such a quantification, the volume occupied by EDDs was measured; then, these number was divided with the reference volume of the cytoplasm. These data showed a significant increase in the calcium level induced by a passive transfer in all ALS-sera-treated mice (Figure 4). Compared to the untreated and healthy-serum-treated controls, all animals showed elevated calcium levels. The elevation of the intracellular calcium level was most prominent in mice treated with ALS sera from patients with hexanucleotide repeat expansions (HNRs) in the *C9ORF72* gene.

### 2.4. Number of Lumbar Motor Neurons Is Reduced after Injection of Amyotrophic Lateral Sclerosis Sera into Mice

**Figure 5:**
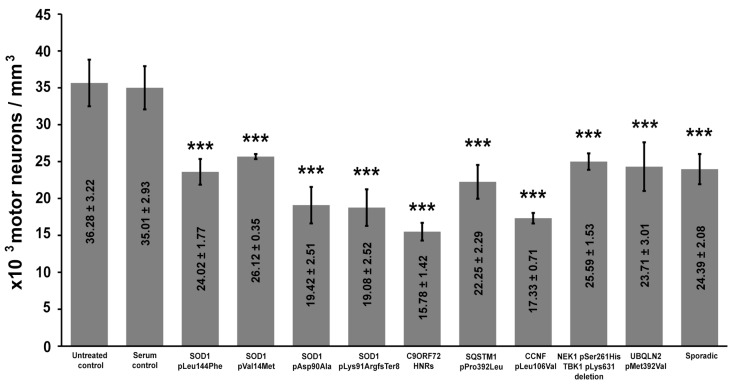
While there was no difference between the untreated and the healthy-sera-treated animals, the number of motor neurons was reduced in all amyotrophic lateral sclerosis sera-treated groups. Data values are displayed within the bars, and represented by mean value ± standard error of the mean. Statistical evaluation was determined using one-way analysis of the variance with the Bonferroni post hoc pairwise comparison. ***: *p* < 0.001.

The number of MNs in the lumbar segment of the spinal cord was determined by the optical disector method and expressed as number/mm^3^. After treatment with sera from ALS patients, all injected animals showed neuronal loss, compared to the untreated controls, which was most prominent in mice injected with sera from patients with the *C9ORF72* mutation (Figure 5). In detail, the number of MNs in the ventrolateral pool of the spinal cord was not changed after a passive transfer of the sera from healthy individuals compared to the untreated control. However, all inoculated groups of animals with different ALS sera showed a reduced number in this anatomical region.

### 2.5. Correlation of Intracellular Calcium Increase in Motor Neurons and Loss of Spinal Motor Neurons of Amyotrophic Lateral Sclerosis Serum-Treated Mice

**Figure 6:**
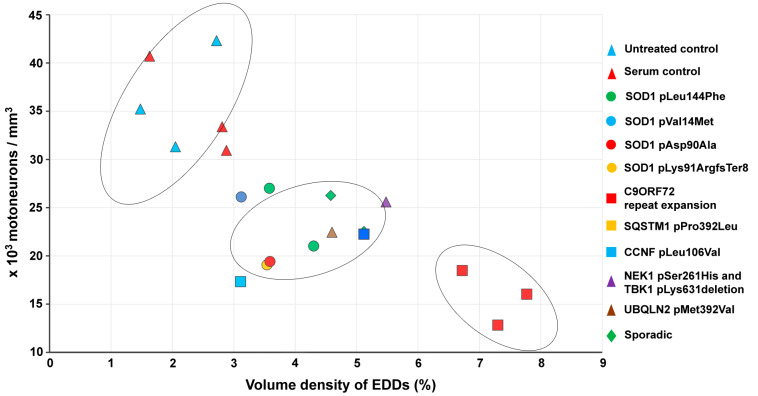
On the diagram (except for the untreated animals), each symbol represents a patient who served as a serum donor. Clearly, by increasing the observed intracellular calcium level (horizontal axis), the decreasing number of surviving motor neurons could be counted (vertical axis). With statistical analysis, three clusters could be identified: the control group at the left side, the majority of amyotrophic lateral sclerosis patients at the middle, and patients with *chromosome 9 open reading frame 72* hexanucleotide repeats, with the most substantial effect, at the right side. For a visual representation, 99.5% confidence intervals were determined for the three clusters, drawn as ellipses around the clusters.

Since the central role of calcium in the degeneration process of MNs is well established [23], a correlation between the treatment-induced elevation of intracellular calcium and the eventual loss of MNs can be implicitly hypothesized. Thus, the cross-relation of the intracellular calcium level of MNs and the number of surviving lumbar MNs after a passive transfer of ALS sera was analyzed by plotting these parameters after pooling the data to individual serum donor patients (Figure 6). The diagram demonstrates that the larger the calcium increase induced by the serum from a particular patient, the smaller the number of surviving MNs in the spinal cord (Figure 6). The data representing the control group were significantly separated from the ALS-serum-treated patients’ data; furthermore, in the ALS-sera-treated group, the data representing the *C9ORF72* HNRs formed a separate subgroup (Figure 6).

### 2.6. Correlation of Intracellular Calcium Increase in Motor Neurons and Motor Axon Terminals of Amyotrophic Lateral Sclerosis Serum-Treated Mice

**Figure 7:**
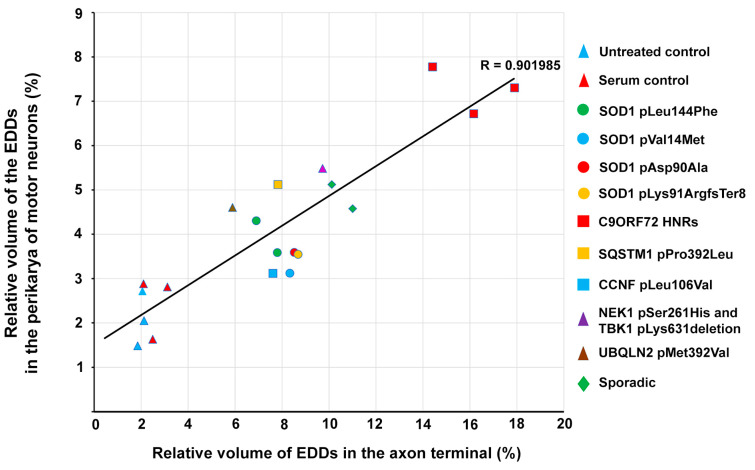
Volume densities of the electron-dense deposits from the two anatomical regions of the spinal motor neurons (MNs) of injected mice were pooled to patients and plotted in such a way that (except for the untreated animals) each symbol represented a patient who served as a serum donor. Passive transfer of amyotrophic lateral sclerosis sera resulted in a mutual increase in calcium levels in the central and peripheral parts of the MNs. This observation was corroborated by the analysis of the fitted linear model, where the correlation coefficient (R) was 0.902, representing a strong correlation between the examined variables.

In our previous study, we demonstrated a significant calcium increase in the axon terminals of lumbar MNs in a similar experimental paradigm as in the present study [21]. To analyze whether analogous changes in the intracellular calcium could be evoked by the ALS serum treatment in the peripherally located motor axon terminals and the centrally located motoneuronal cell bodies, calcium data from these anatomical regions were pooled to patients serving as serum donors, plotted (Figure 6), and evaluated using a linear regression analysis. The statistical evaluation showed a strong correlation (R = 0.902) between the elevation of the calcium level in the perikarya and axon terminals of lumbar MNs represented by the volume density of EDDs (Figure 7). This correlation indicates that ALS sera initiated a similar elevation in the calcium level in the MNs and their distant axon terminals after a passive transfer compared to untreated and healthy-sera-treated controls.

## 3. Discussion

The implication that circulating toxins might be responsible for the degeneration of the anterior horn cells was based on the seminal observation of Wolfgram and Myers describing that diluted serum, specifically from ALS patients, had a toxic effect on mouse anterior horn cells in culture [24]. From the efforts to model sporadic ALS based on circulating agents, those based either on the effect of cerebrospinal fluids [25,26] or of sera from the patients [27] can be emphasized. Indeed, a line of research data from Appel’s laboratory documented the presence of immunoglobulins in spinal and cortical motor neurons [28] and the ability of immunoglobulins from sporadic ALS patients (i) to induce apoptotic cell death in a hybrid motoneuron cell line [29], (ii) to increase the Ca^2+^ current [30] and calcium level in motoneurons in vitro [31], and (iii) to increase the intracellular calcium of cell bodies and axon terminals of MNs and the increase in synaptic vesicles at the neuromuscular synapses, in vivo [19]. The pathobiological relevance of these findings received convincing support by delicate structural and microanalytical data displaying comparable alterations to human motor axon terminals in muscle biopsy samples obtained from sporadic ALS patients [17]. The detrimental effect of ALS-specific antibodies in in vitro and in vivo studies is well-known. Still, in the case of a systemic administration, the transport mechanisms behind how purified IgG or sera components migrate to the central nervous system are poorly understood. One possible explanation might be the disruption of the blood–brain barrier and blood–spinal cord barrier, the native barriers between the central nervous system and the circulatory system, which are indeed altered in ALS [32,33]. This complex anatomical structure has a crucial role in neurodegeneration and potential immunotherapies [34,35]. ALS mouse models showed increased permeability of microvessels for Evans blue at the early stage of the disease [36]. An impairment of the neurovascular unit was demonstrated in the ALS rat model by the decreased expression of tight junction proteins of endothelial cells as well as zonula occludens and claudin proteins in the spinal cord [37,38]. Such disruption in the barrier properties promotes the migration of immunoglobulins [39]; furthermore, the recruitment of immunocompetent cells at the motor axon terminal also supports the migration of antibodies from the periphery [40]. Such a degeneration of the neuromuscular junction was noted in the mice used in this study (Figure S3) and our previous study after a long-term inoculation with ALS sera [20].

The availability of sera from patients with no family history of the disease made it possible to quantitatively characterize the pathological and microchemical abnormalities and the reduced physical performance induced by the injection of the sera into mice [20]. In two weeks, a repeated injection of ALS serum induced an approximate 60% loss of MNs at the lumbar section of the spinal cord, accompanied by a 60% loss of physical performance expressed by the inability to hang on a vertical grid and significant increases in the intracellular calcium of surviving spinal MNs and in the motor axon terminals, measured with an electron microscopic histochemical method [20].

Next, to examine whether comparable changes could be induced with sera from ALS patients with different identified mutations, including those with a documented family history, were selected as serum donors. Meanwhile, healthy individuals, and patients with negative screening results for all 35 major ALS genes, regarded as sporadic ALS patients, served as the control. Using a short-term repeated injection paradigm, an increased synaptic vesicle density and increased calcium could be detected in the motor axon terminals of the injected mice [21], similar to those changes detected after the injection of sera from sporadic ALS patients [20] or seen in muscle biopsy samples of sporadic patients [17]. Furthermore, on the scatter diagram vesicle number and intracellular calcium, the population of ALS patients could be separated from the controls [21], again, similarly as in the case of human samples [17].

To answer whether comparable changes could be induced in the cell bodies of the spinal MNs as in the axon terminals with the injections of these sera, in the present experiments, the injections were repeated in the same way as previously using a new set of animals. Afterward, the cell number and the calcium content of MNs in the lumbar spinal cord were analyzed. First, a decrease in the number of MNs was observed with increased intracellular calcium in the surviving population. Interestingly, in the scatter plot of these parameters, the population of ALS patients could be separated from the controls, similarly as in the case of the axon terminals [21]. Secondly, the calcium increase in the motor axon terminals showed a strong correlation with that of the cell bodies, indicating that analogous changes could be induced in these distant anatomical regions of MNs with the injection of the same ALS sera. These passive transfer experiments accentuated the similar central role of calcium in the process of sporadic and familial ALS patients, regardless of the primary etiological cause of the disease, which might be at least partially responsible for the clinically similar course of ALS. The general role of calcium in the pathomechanism of ALS was further supported with the findings obtained in the commonly used SOD1 transgenic model of ALS: a similar motoneuronal loss in the spinal cord with increased calcium could be observed [18], as documented in the present study.

For the present experiments, instead of purified IgG, sera from ALS patients were used for the inoculation, as in our previous studies [20,21], because it demonstrates more complex and more substantial biological effects than the isolated IgG. However, the presence of immunoglobulins was demonstrated in the spinal MNs. The degenerative effect of ALS serum might come from multiple pathways. On the one hand, it comes from the presence of numerous autoimmune IgG antibodies, characterized by the examination of sera from ALS patients [41]. Besides the autoimmune properties, these antibodies can elevate the motoneuronal calcium level [42,43] and induce selective MN apoptosis in rat mixed primary spinal cord cultures [44]. Immunoglobulins from patients with sporadic ALS can bind and modify the function of L-type, P-type, and other neuronal calcium channels [45,46,47], which increase the frequency of miniature end-plate potentials eventually [30]. ALS-specific immunoglobulins from ALS patients or immunized goats also increased tumor necrosis factor α, interleukin-6, and interleukin-10 levels in the spinal cord, which are well-known proinflammatory factors in mice after intraperitoneal administration [48]. The importance of these autoantibodies in the pathobiology of ALS is further accentuated by the fact that by neutralizing these antibodies, the pathobiological effects diminish [30]. Admitting that the subsets of spinal MNs may have different susceptibility [49] to answer whether it can be attributed to their unique intrinsic properties, e.g., the calcium-binding protein content [50,51] or different ability to uptake IgG, warrants further studies.

The large extent of motoneuronal loss in the present experiments seemed to be surprising; however, numerous spinal cord injury models showed a fast and significant loss of MNs. For example, 44% of neurons diminished after one day after dorsal compression, which peaked at day 3 when almost 75% of the neurons were lost [52]. After spinal transection injury, a rapid neuronal loss was observed 12 h following injury [53]. Similar to these models, MNs showed a rapid degeneration after a couple of hours in contusion, compression, and transection models [54,55]. Besides the surgical methods, ischemic injury can initiate similarly fast neuronal degeneration as well [56]. Furthermore, a fast and significant loss of MNs could be induced in rat pups with intrathecal injection of ALS cerebrospinal fluid [26].

In the present study, we demonstrated that, based on the correlation of the cell loss and the calcium increase, ALS patients could be sorted into clusters different from the controls as in the case of data obtained in the motor axon terminals [21]. Moreover, a subgroup of patients with the *C9ORF72* mutation could also be identified. The remarkable effect of this mutation might be attributed to a mixture of the loss of function and a toxic gain of function of the C9ORF72 protein, along with a strong immune/inflammatory effect [57,58,59].

In summary, we demonstrated that ALS-like pathological alterations could be induced in the spinal MNs and axon terminals of the injected animals by injecting the patients’ sera regardless of the known etiological cause of the disease. The induced degeneration of the MNs accompanied by the calcium increase points to the central role of calcium in the pathomechanism of the disease, which may be the mediator of a common final pathway of this degenerative disease [23].

## 4. Materials and Methods

### 4.1. Ethical Approvals and Consent to Participate

The protection and welfare of animals were carried out according to the national law (Edict 40/2013 (II.14)) which conforms to the European Union executory directive on the protection of animals used for scientific purposes (Directive 2010/63/EU amended by the Regulation (EU) 2019/1010). Ethical approvals for the scientific experiments involving animals were given by The Government Office in Csongrád-Csanád County, Hungary (#XI/4962/2015; #XVI/819/2021) and by The Committee for Animal Experiments of the University of Szeged, Szeged, Hungary (I. 74-II/2015) and by the Ethical Committee for the protection of Animals in Scientific Research at the Biological Research Center, Szeged, Hungary (#XVI/819/2021). All efforts were made to minimize animal suffering throughout the experiments. Ethical approvals for obtaining and storing blood samples for research purposes from ALS patients and healthy controls in an anonymous manner were given by The Human Investigation Review Board, University of Szeged, Hungary (#2557/2009). All experiments using human samples in our project agreed with the declaration of the Medical World Federation proclaimed in Helsinki 1964.

### 4.2. Patients

The same ALS patients and three healthy controls participated in this study as in our previous work [21]. Among these individuals, five ALS patients possessed mutations in the *SOD1* gene. Three patients had GGGGCC HNRs in the *C9ORF72* gene. One patient had a mutation in the *SQSTM1* gene, another one had a mutation in the *CCNF* gene, a further one had a mutation in the *UBQLN2* gene, and, finally, a patient with ALS and frontotemporal dementia had a double mutation in the *NEK1* gene together with a mutation in the *TBK1* gene, respectively. Two patients, composing the sporadic ALS group, were genetically screened for all 35 major ALS genes and proved to be negative. For controls, sera from 3 age-matched healthy volunteers were obtained.

### 4.3. Passive Transfer of the Human Sera and Tissue Preparation for Electron Microscopy

Forty-eight male Balb/c mice, obtained from Charles River Appoints AnimaLab Hungary Kft. (Vác, Hungary), were injected intraperitoneally with 1 mL/day serum from ALS patients with different mutations (*n* = 3) or healthy individuals (*n* = 3) for two days. All animals received sera from the same patient during the inoculation period. Afterward, animals treated with sera with the same mutation were pooled together since there was no statistical difference between the effect of the sera from different patients with the same mutation. Therefore, the grouping of the experimental animals was based on the mutations, not the patients. A similar pooling protocol was applied to animals treated with sera from sporadic patients. One group of animals did not receive an injection and was used as the untreated control (*n* = 3).

During the two-day inoculation period, animals were housed in plastic cages (5 animals/cage, at most) in a thermoneutral environment (22 ± 3 °C) and reversed 12 h light/dark cycle with access to water and regular rodent chow ad libitum. Twenty-four hours after the last serum injection, lumbar spinal cords were dissected from the animals for the electron microscopic evaluation of intracellular and suborganellar localization of calcium in tissue samples as described in our previous study [21].

### 4.4. Immunohistochemical Demonstration of Human Immunoglobulins in the Lumbar Neurons

To illustrate the presence of human immunoglobulins in spinal neurons, mice after passive transfer of sera from ALS patients or healthy individuals, or without any treatment, were anesthetized with Avertin, then transcardially perfused with 10 mM phosphate-buffered saline (PBS), followed by 4% paraformaldehyde (Σ-Aldrich, St. Louis, MO, USA; pH adjusted to 7.4 with NaOH). L2–L5 lumbar spinal cord segments were postfixed in the same fixative for 24 h; then, tissue samples were cryoprotected in 30% sucrose solution. After all spinal cord tissue was submerged in the solution at 4 °C, 30 µm thin sections were prepared on a freezing microtome (Reichert-Jung, Wetzlar, Germany). Free-floating sections were rinsed in 10 mM PBS for 5 min (3 times); then, 2% normal goat serum in 10 mM PBS with 0.2% Triton X-100 (TPBS) was used for 60 min to block non-specific binding sites. Afterward, samples were incubated with rabbit anti-choline-acetyltransferase antibody (Thermo Fisher Scientific, Waltham MA, USA) diluted to 1:1000 in TPBS containing 2% normal goat serum at 4 °C, overnight. After rinsing samples in 10 mM PBS for 5 min (3 times), goat anti-human Alexa488 (Thermo Fisher Scientific) antibody diluted to 1:500 in TPBS containing 2% normal goat serum for 1 h was used to visualize the infiltrated human immunoglobulins from the sera into spinal neurons. Before incubating the samples with goat anti-rabbit Alexa Fluor 546 (Thermo Fisher Scientific) diluted and incubated identically to the other secondary antibody, samples were washed in 10 mM PBS for 5 min (3 times). Finally, stained samples were rinsed in 10 mM PBS for 5 min. Afterward, all samples were mounted on silane-coated glass slides, covered with Fluoromount-G medium (Sigma-Aldrich), and visualized under a confocal microscope (Eclipse C1, Nikon, Tokyo, Japan). All digital images were uniformly processed with the two-dimensional blind deconvolution module of the AutoQuant X program (version X2.2, Media Cybernetics, Rockville, MD, USA); then, contrast and brightness were modified as needed to improve image quality and reduce background noises.

### 4.5. Counting the Lumbar Motor Neurons on Histological Sections

After fixation, segments from the intumescence of the lumbar spinal cords were spared for quantification of motoneuronal survival in the ventrolateral MN pools. As in our previous experiments, the lumbar section was chosen for evaluation since hindlimb weakness is one of the first symptoms of MN degeneration in immune-mediated and transgenic animal models [20,21]. Sections were placed in a 30% sucrose solution. After cryoprotection, consecutive 10 µm thin sections were cut with a cryostat (Reichert-Jung, Leica Biosystems, Wetzlar, Germany) and stained with cresyl violet. Estimation of the number of MNs was performed using the optical disector method on the cresyl-violet-stained samples. Images were recorded and examined with the Image-Pro Plus image analysis program (version 7.0.0.591, Media Cybernetics) and a MicroPublisher 5.0 RTV charge-coupled device camera (QImaging, Surrey, BC, Canada) attached to an Eclipse 80 i (Nikon) light microscope. The MNs were identified by their size, location in the ventrolateral position, and their ultrastructural features. The anatomical border of the ventrolateral pool of MNs was determined using the method described by Kong et al. [60]. The cells were counted if they met the selection criterion of the disector, which was the presence of the nucleolus in the section in either optical plane. Counts from consecutive sections (*n* = 8/animal) were pooled for animals, and the average numbers of MNs in the lumbar segments were determined for each group.

### 4.6. Quantification of the Intracellular Calcium Level in the Spinal Motor Neurons

Sections were screened for the presence of the profiles of MNs in the ventral horns of the lumbar spinal cord, and images were recorded in a JEM-1400 Flash transmission electron microscope (JEOL, Tokyo, Japan). The relative volume of the perikarya occupied by the EDDs representing the calcium precipitates was determined, as described previously in the work of Meszlényi et al. [21].

### 4.7. Statistical Analysis

To determine the average volume occupied by EDDs within the cellular compartments of MNs, the data derived from individual electron microscopic fields were pooled according to animals and passive transfer groups. From each animal, 15-15 perikaryal areas were analyzed on the TEM images of these MNs. The number of MNs on both sides of the lumbar spinal cord was determined on cresyl-violet-stained samples (*n* = 8/animal), and values were averaged per animal. Differences among multiple means of the volume density of the EDDs were assessed by the one-way analysis of variance, followed by the Bonferroni post hoc test. Expectation–Maximization cluster analysis was performed with WEKA (version 3.8.3, Waikato, New Zealand) to investigate the cross-relation of the volume density of EDDs and the loss of MNs in patients with different ALS genotypes and controls. All statistical analyses were performed with R (version 3.6.2, R Foundation for Statistical Computing, Vienna, Austria) with R Studio Integrated Development Environment (version 1.1.453, RStudio Inc., Boston, MA, USA) for Windows. All data are presented as mean values ± the standard error of the means (s.e.m.).

## Data Availability

All data are presented within the manuscript or available by request to one of the corresponding authors, Roland Patai, (patai.roland@brc.hu) or László Siklós (siklos.laszlo@brc.hu).

## Supplementary Materials

The following are available online at https://www.mdpi.com/article/10.3390/ijms22189994/s1.

## Abbreviations

ALS	Amyotrophic lateral sclerosis
C9ORF72	Chromosome 9 open reading frame 72
*CCNF*	G2/mitotic-specific cyclin F
EDDs	Electron-dense deposits
HNRs	Hexanucleotide repeat expansions
IgG	Immunoglobulin G
MN	Motor neuron
*NEK1*	Never in a mitosis A-related kinase 1
PBS	Phosphate-buffered saline
SOD1	Superoxide dismutase 1
*SQSTM1*	Sequestosome 1
*TBK1*	TANK-binding kinase 1
TPBS	Phosphate-buffered saline with 0.2% Triton X-100
*UBQLN2*	Ubiquilin 2
