# Supplementary figures and images for: Passive Transfer of Blood Sera from ALS Patients with Identified Mutations Results in Elevated Motoneuronal Calcium Level and Loss of Motor Neurons in the Spinal Cord of Mice
Source: Int J Mol Sci. 2021 Sep 16;22(18):9994. doi: 10.3390/ijms22189994 (PMC8470779; doi:10.3390/ijms22189994)

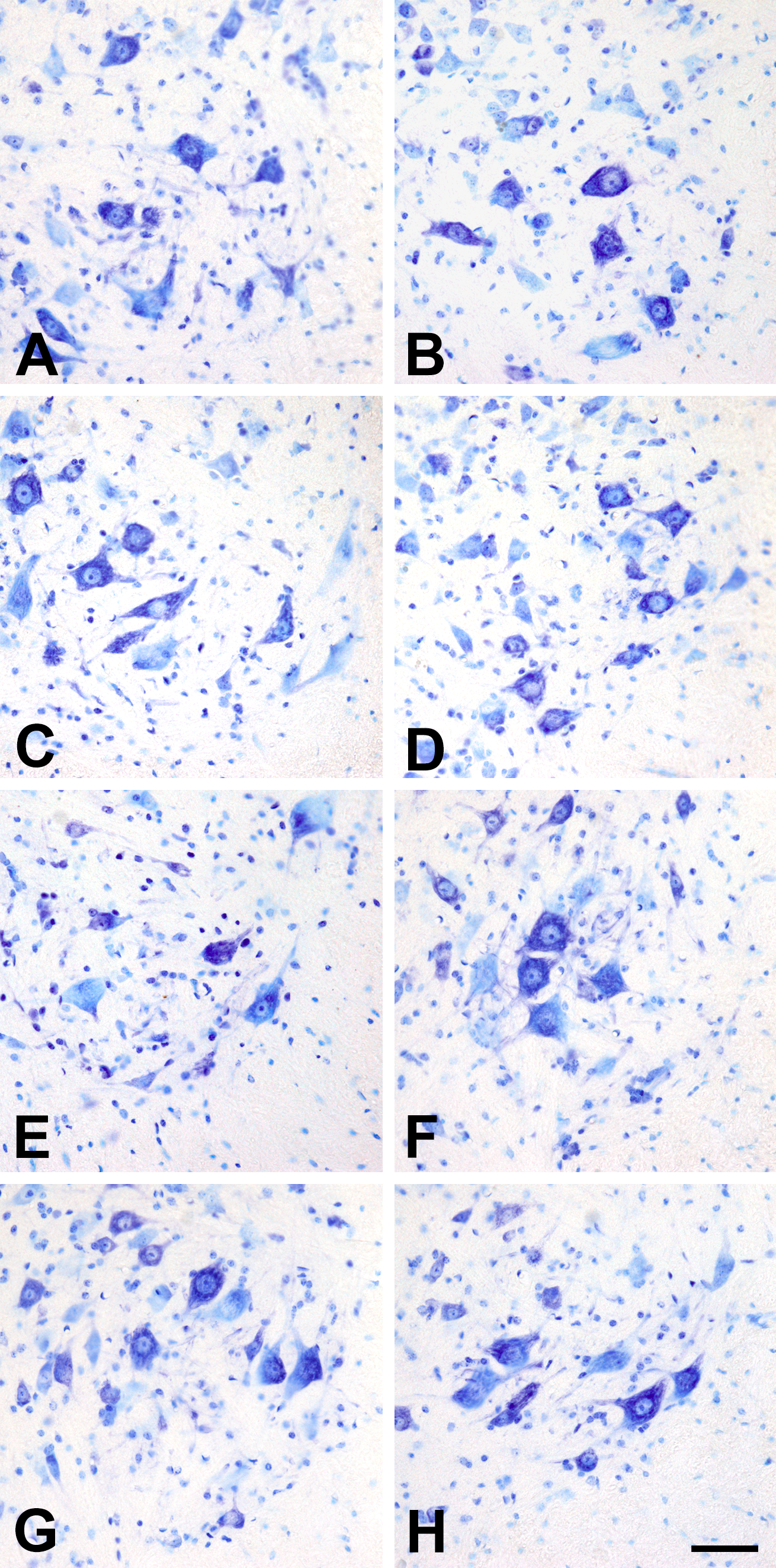

Supplement: Supplementary file 1 [file ijms-22-09994-s001.zip › Supplementary_figure_1.tif]

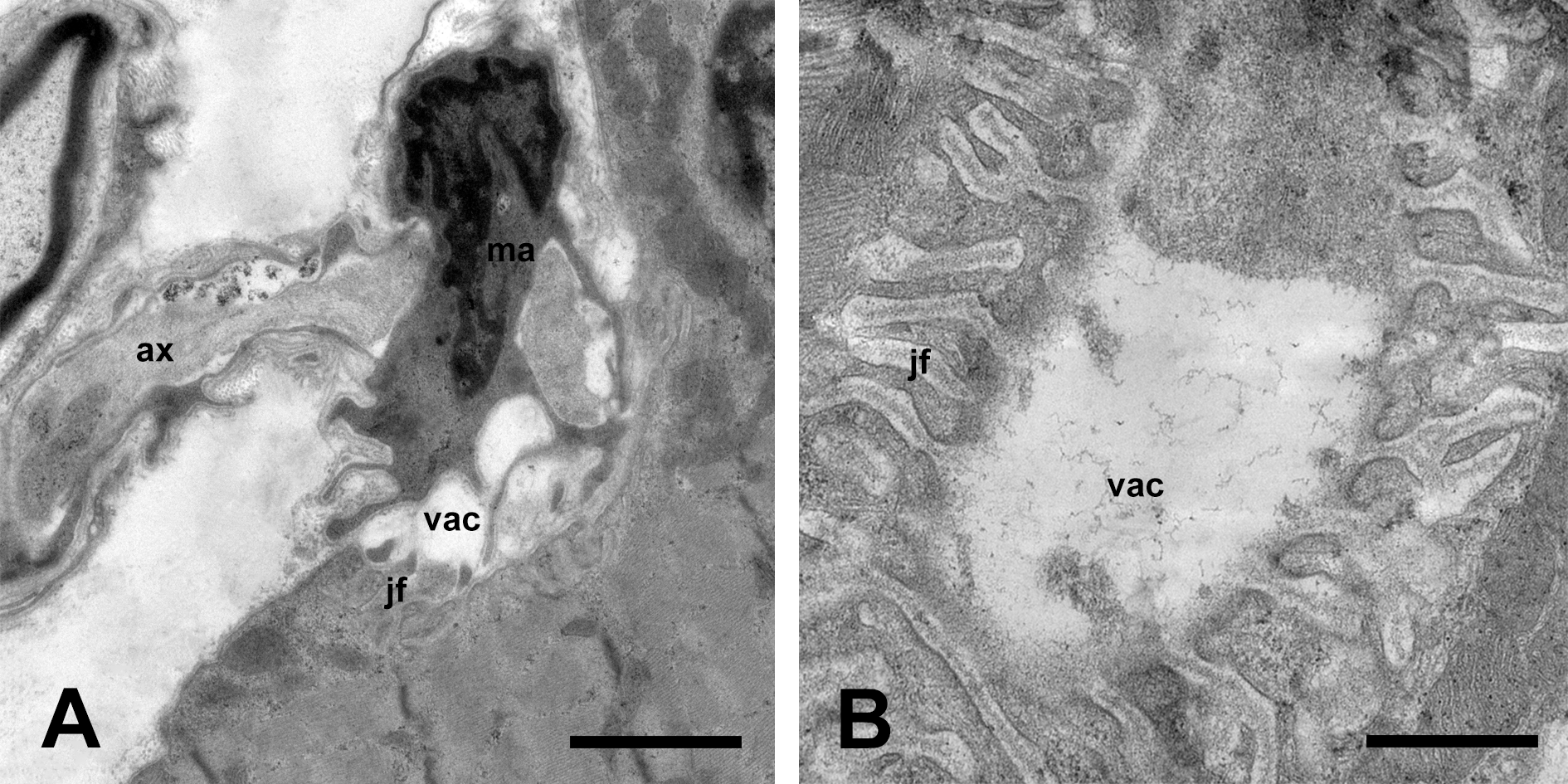

Supplement: Supplementary file 1 [file ijms-22-09994-s001.zip › Supplementary_figure_3.tif]

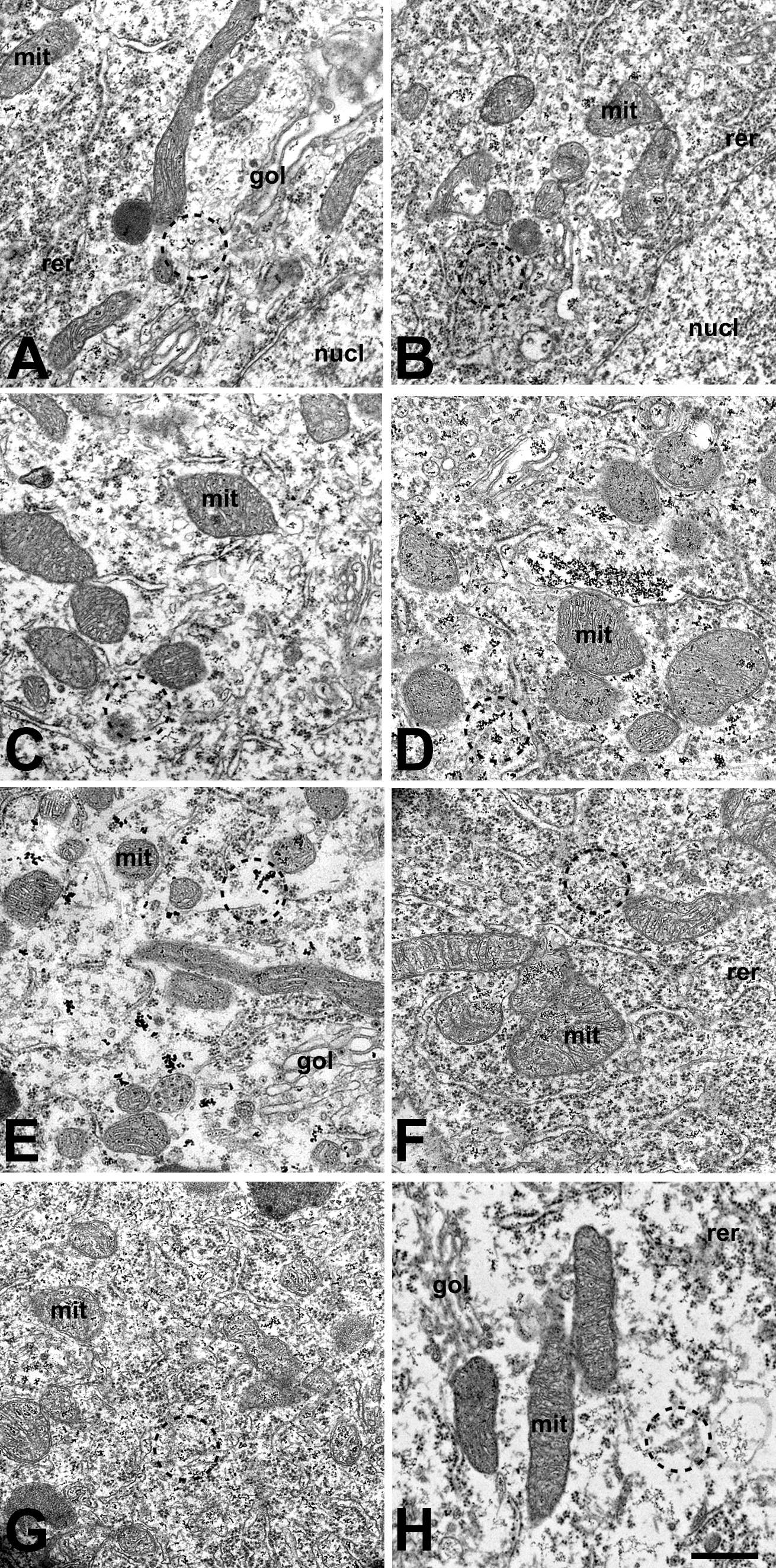

Supplement: Supplementary file 1 [file ijms-22-09994-s001.zip › Supplementary_figure_2.tif]
